# Complete Genome Sequence of Escherichia coli O157:H7 Strain Al Ain, Isolated from Camel Feces in the United Arab Emirates

**DOI:** 10.1128/MRA.01171-19

**Published:** 2019-11-14

**Authors:** Dawood Al-Ajmi, Shafeeq Rahman, Sharmila Banu

**Affiliations:** a College of Food and Agriculture, Department of Integrative Agriculture, UAE University, Al Ain, United Arab Emirates; Loyola University Chicago

## Abstract

Escherichia coli O157:H7 is a common food pathogen which has a serious effect on human health. We report here the complete genome sequence of Escherichia coli O157:H7 strain Al Ain, isolated from camel feces in the United Arab Emirates.

## ANNOUNCEMENT

Enterohemorrhagic Escherichia coli O157:H7 (E. coli O157) is a human pathogen transmitted through the consumption of contaminated foods, such as beef and dairy products, vegetables, and fruits (1, 2). E. coli O157 belongs to the larger category of Shiga toxin-producing E. coli (STEC), which has the ability to produce Shiga toxin type 1 (*stx*_1_), Shiga toxin type 2 (*stx*_2_), or both toxins along with other variants (3, 4). E. coli O157:H7 is responsible for severe abdominal illness, specifically enterohemorrhagic colitis and hemolytic uremic syndrome, and usually causes severe diarrhea (5). Healthy cattle are the main reservoir of STEC, although it is also carried by other animals (6). Based on previous works, it was generally concluded that E. coli O157 was not present in camel feces in the United Arab Emirates (UAE) (7, 8). However, these results were based mainly on the characterization of a very few colonies picked from the sample and lacked a specific screening and isolation technique. We sequenced E. coli O157:H7 to better understand colonization in camels, which will also help us develop more effective preharvest food safety practices to reduce food contamination in the slaughterhouse.

Approximately 10 cm of the rectoanal junction was cut immediately after slaughter; the fecal samples were collected, kept refrigerated (4°C), and transported to the laboratory, where they were examined. Microbial testing was performed within 3 h of collection. An enriched fecal sample (1 ml) was mixed with 20 μl of magnetic beads coated with O157 antibody, and immunomagnetic separation (IMS) was performed according to the manufacturer’s instructions (Oxoid, UK). The bead suspension (100 μl) was streaked onto two plates of McConkey sorbitol agar with cefixime-tellurite (CT-SMAC, Oxoid). The plates were incubated at 37°C for 24 h, and pure E. coli O157 colonies were identified. A single colony was picked and further grown in LB broth (HiMedia) at 37°C for 18 to 24 h with constant shaking (200 rpm). Genomic DNA was extracted from strain O157:H7 using a bacterial genomic DNA isolation kit (Norgen Biotek, Canada), and the colonies were further confirmed using *E. coli* O157-specific PCR primers according to Desmarchelier et al. (9).

DNA library preparation was carried out using a SMRTbell template prep kit (Pacific Biosciences), and the library was sequenced using the PacBio RS II platform (Macrogen, South Korea). A total number of 185,287 reads with a mean subread length of 8,028 bases (*N*_50_, 12,742 bases) were obtained. Approximately 100% genome coverage was observed with 200× sequencing depth. *De novo* assembly was performed using the Hierarchical Genome Assembly Process v3.0 (HGAP3) with default parameters (10) within SMRT Analysis v2.3.0 software. Genome annotation was carried out using the NCBI Prokaryotic Genome Annotation Pipeline v4.8 (PGAP) (11). Using one single-molecule real-time (SMRT) cell on the PacBio RS II sequencing platform, we obtained 5,444,610 bp containing 5,399 coding sequences (CDSs), 105 tRNAs, 22 rRNAs, and 8 noncoding RNAs (ncRNAs). The A+T content was 49.7%, and the G+C content was 50.3%.

In-depth analyses of these isolates are in progress and will provide more information regarding *E. coli* O157 and its virulence genes in camels. Furthermore, understanding the evolution of these particular strains in relation to other isolates will help us understand more about these strains.

### Data availability.

The genome sequence of E. coli O157 strain Al Ain has been deposited in the NCBI GenBank database under accession number CP043539. The raw sequences are available in the NCBI SRA database under accession number SRR10127193. The versions described in this paper are the first versions.
